# Dexpanthenol ameliorates lipopolysaccharide-induced cardiovascular toxicity by regulating the IL-6/HIF1α/VEGF pathway

**DOI:** 10.1016/j.heliyon.2024.e24007

**Published:** 2024-01-05

**Authors:** Mustafa Soner Ozcan, Mehtap Savran, Duygu Kumbul Doguc, Hatice Kubra Dogan, Melike Altintas, Samet Cosan

**Affiliations:** a Department of Anesthesiology and Reanimation, Faculty of Medicine, Suleyman Demirel University, Isparta, Turkey; b Department of Pharmacology, Faculty of Medicine, Suleyman Demirel University, Isparta, Turkey; c Department of Biochemistry, Faculty of Medicine, Suleyman Demirel University, Isparta, Turkey; d Department of Bioengineering, Institute of Science, Suleyman Demirel University, Isparta, Turkey; e Department of Pathology, Faculty of Veterinary Medicine, Burdur Mehmet Akif Ersoy University, Burdur, Turkey

**Keywords:** Lps, Sepsis, Pantothenic acid, Inflammation, Oxidative stress

## Abstract

**Introduction:**

Lipopolysaccharide (Lps) is an essential component responsible for the virulence of gram-negative bacteria. Lps can cause damage to many organs, including the heart, kidneys, and lungs. Dexpanthenol (Dex) is an agent that exhibits anti-oxidative and anti-inflammatory effects and stimulates epithelialization. In this study, we aimed to investigate the effects of Dex on Lps-induced cardiovascular toxicity.

**Methods:**

Rats were divided into four groups: control, Lps (5 mg/kg, intraperitoneal), Dex (500 mg/kg, intraperitoneal), and Lps + Dex. The control group received saline intraperitoneally (i.p.) once daily for three days. The Lps group received saline i.p. once daily for three days and a single dose of Lps i.p. was administered on the third day. The Dex group received Dex i.p. once daily for three days and saline on the third day. The Lps + Dex group received Dex i.p. once daily for three days and a single dose of Lps i.p. on the third day. Heart and aortic tissues were taken for biochemical, histopathological, immunohistochemical, and genetic analysis.

**Results:**

Lps injection caused histopathological changes in both heart and aortic tissues and significantly increased total oxidant status and oxidative stress index levels. Interleukin-6, and Tumor necrosis factor-α mRNA expressions were significantly altered in heart and aorta, likely do to the anti-inflammatory and antioxidative effects of Dex. Furthermore, Dex affected Caspase-3 and Hypoxia-inducible factor 1-α staining patterns.

**Conclusions:**

Our results show that Dex treatment has a protective effect on Lps-induced cardiac and endothelial damage in rats by reducing inflammation, oxidative stress, and apoptosis.

## Abbreviations

Lps: Lipopolysaccharide
TLR4: Toll-like receptor 4
TNF-α: Tumour necrosis factor- α
IL-6: Interleukin-6. ROS=Reactive oxygen species
HIF1: Hypoxia-inducible factor 1
VEGF: Vascular endothelial growth factor
Dex: Dexpanthenol. Con = Control
i.p: Intraperitoneally
HE: Hematoxylin eosin
PBS: Phosphate-buffered saline
Cas-3: Caspase-3
CK-MB: Creatine kinase myocardial band
LDH: Lactate dehydrogenase
TAS: Total antioxidant status
TOS: Total oxidant status
OSI: Oxidative stress index
ABTS: 2,2ʹ-azino-bis (3-ethylbenzthiazoline-6-sulfonic acid)
RT-qPCR: Reverse transcription-quantitative polymerase chain reaction

## 1 Introduction

Sepsis, ‘’life-threatening organ dysfunction caused by a deregulated host response to infection’’, is the most common cause for intensive care unit admissions and causes dysfunction in the heart, blood vessels, lungs, liver, and kidneys [1,2]. In 2017, sepsis was responsible for approximately 11 million deaths (19.7 %) worldwide [3].

The adult human heart beats an average of 60–100 times per minute in a normal sinus rhythm. Irregularities of the cardiovascular system, such as occlusions in the coronary arteries, insufficiency in the heart valves, rhythm disorders, or inflammation in the heart muscle, can limit the routine daily activities of people, can result in hospitalization to intensive care units, and even death. Due to uncontrolled inflammation in the heart, cells can undergo apoptosis and heart failure can occur [4]. Cardiac inflammation can be local or caused by systemic inflammation, i.e. sepsis.

Sepsis-induced myocardial dysfunction (SIMD) is a reversible condition associated with poor prognosis in patients with sepsis [5,6]. Although validated studies are lacking, standard therapeutic approaches, including vasopressors, inotropes, and fluid resuscitation have been used to treat SIMD [7,8]. Despite many inflammatory pathways reportedly involved in the pathogenesis of SIMD and increasing evidence regarding alternative treatment methods, more data are needed to make definitive recommendations for clinically targeting inflammatory pathways [7,9].

Inflammatory responses caused by sepsis can be mimicked with lipopolysaccharide (Lps), which is responsible for the virulence of gram-negative bacteria, as shown in animal studies [10]. Lps endotoxins activate Toll-like receptor 4 (TLR4) in human cells [11] stimulating pathways that trigger each other and initiate damage mechanisms, such as oxidative stress, inflammation, and apoptosis [12]. As a result, pro-inflammatory cytokines including Tumor necrosis factor-α (TNF-α) and Interleukin-6 (IL-6) are secreted from the affected cells [13]. Reactive oxygen species (ROS) are generated in cardiac cells that have inflammation, resulting in oxidative stress in the cardiac tissue [14]. Cells undergo apoptosis or necrosis if oxidative stress-mediated damage is not suppressed. To prevent the occurrence of such damage, specific intracellular pathways can be activated to promote vascularization of the affected tissue.

**Fig. 1:**
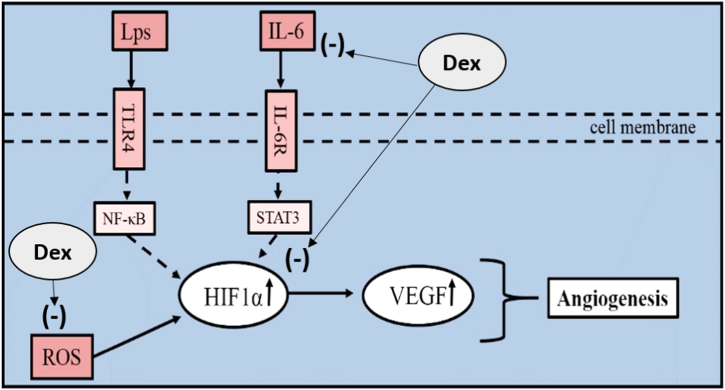
The potential action mechanism of the HIF1α/VEGF pathway.

Hypoxia-inducible factor 1 (HIF1) is a heterodimeric transcription factor consisting of two subunits (HIF1α and HIF1β). Oxygen-sensitive HIF1α degrades rapidly under normocytic conditions; however, hypoxic conditions stabilize the expression of HIF1α [15,16]. HIF1α, which is promoted by ROS and IL-6, stimulates angiogenesis by increasing Vascular endothelial growth factor (VEGF) levels (Fig. 1) [17].

Dexpanthenol (Dex) is a biologically active analogue of pantothenic acid that exhibits anti-oxidative and anti-inflammatory effects and stimulates epithelialization [18]. Dex has been reported to have protective effects in several organs [[19], [20], [21]]. Furthermore, the antioxidative and anti-inflammatory features of Dex were highlighted as potential ameliorative mechanisms in various cardiac injury models [[22], [23], [24]]. This study aimed to evaluate the effect of Dex on Lps-mediated inflammation in rat heart tissue with regards to the IL-6/HIF1α/VEGF pathway.

## 2 Materials and methods

### Ethical approval

All experimental procedures were performed according to National Institutes of Health animal research guidelines and with approval from the Committee on Animal Research of Suleyman Demirel University, Isparta (Ethic No: September 15, 2022/06–71). The Scientific Research Projects Coordination Unit of Suleyman Demirel University supported this study (TSG-2020-8134).

### 2.1 Study design

Due to their size and anatomy to create a sepsis model, male Wistar Albino rats (n = 32, 250–350 g) were used in the study. A rat model was also preferred because of the availability of large databases for comparisons with previously obtained data related to human sepsis. Rats were kept under standard conditions (21°C-22 °C temperature, 60 % ± 5 % humidity, 12:12 h light/dark cycle) and given a standard commercial chow diet (Korkuteli Yem, Antalya, Türkiye). Rats were randomly assigned to one of four groups, with eight rats per group:

**Control (Con):** Saline (1 ml) was administered intraperitoneally (i.p.) into the left inguinal region once daily for three days, and saline (1 ml) was given via the right inguinal region on the third day.

**Lps (5 mg/kg):** Saline (1 ml) was administered i.p. into the left inguinal region once daily for three days. On the third day to create an inflammatory state, a single dose of Lps (048K4126, Sigma Aldrich, USA) dissolved in saline, was given i.p. into the right inguinal region [25].

**Lps + Dex:** 1 ml Dex (500 mg/kg; Bepanthene vial, Bayer AG, Leverkusen, Germany) was administered i.p. into the left inguinal region once daily for three days. Dex was selected because of reported anti-inflammatory and antioxidative features, as a drug to potentially counter the effects of Lps-mediated inflammation [26]. On the third day, a single dose of Lps (5 mg/kg) was administrated i.p. into the right inguinal region.

**Dex:** 1 ml Dex (500 mg/kg) was administrated i.p. into the left inguinal region once daily for three days. Saline (1 ml) was injected into the right inguinal areas on the third day.

Animals were anesthetized with a cocktail of Ketamine (80–100 mg/kg, Alfamin, ALFASAN IBV, Netherlands) and Xylazine (8–10 mg/kg, Alfazin, Alfasan IBV, Netherlands) 6 h after Lps injection. All groups of rats were sacrificed at the same time. Blood samples were taken from the inferior vena cava. The right halves of the heart tissues (including a complete ventricle) and the 1/2 proximal section of the descending aorta were reserved for histopathological analysis, while the left halves of the heart tissues and the remaining parts of the aorta were reserved for biochemical and genetic analyses.

### 2.2 Histopathological analysis

Heart and aorta tissues were fixed in a 10 % buffered formalin solution. The tissue samples were processed and embedded in paraffin, and 5 mm thick sections were cut. After drying, the sections were moved through alcohol and xylol series for dehydration. The sections were stained with hematoxylin and eosin (H&E) then mounted with a coverslip. The sections were examined using a light microscope.

### 2.3 Immunohistochemical analysis

Paraffin-embedded tissue was sliced, dewaxed, dehydrated, and incubated with the primary antibodies of Caspase-3 (Cas-3; E−8; sc-7272; Santa Cruz, Texas, USA) and VEGF (JH1212; sc-57496; Santa Cruz, Texas, USA), at 1/100 dilution, for 60 min. After washing with phosphate-buffered saline (PBS), the sections were incubated with biotinylated secondary antibody (EXPOSE Mouse and Rabbit Specific HRP/DAB Detection IHC kit; ab80436; Abcam, Cambridge, UK) and streptavidin-alkaline phosphatase conjugate. The antigen dilution solution was used as a negative control instead of a primary antibody. Single blinding was performed for all analyses. Semiquantitative analyses were performed to evaluate the markers. The grading scores ranged from 0 to 3 as follows: 0 = negative, 1 = focal weak staining, 2 = diffuse weak staining, and 3 = intense diffuse staining. Ten different areas of each section were examined under 40x magnification, and the Database Manual Cell Sens Life Science Imaging Software System (Olympus Co., Tokyo, Japan) was used for microphotography.

### 2.4 Biochemical analysis

Blood samples were transferred into gel-coated tubes and centrifugated. The levels of creatine kinase myocardial band (CK-MB) and lactate dehydrogenase (LDH) in these samples were measured using an automated analyzer (Beckman Coulter AU 5800, USA).

To analyze oxidative stress in the heart and aorta, 5x (w/v) PBS, at 10 mM pH 7.4, was added to samples for dilution, and tissues were lysed by a homogenizer (Janke&Kunkel IKA Ultra Turrax T25, Germany). Samples were then centrifuged at 4 °C and 2000 rpm for 20 min. Total antioxidant status (TAS) and total oxidant status (TOS) levels of the supernatants were measured using the biochemistry autoanalyzer. According to TAS and TOS measurements, the oxidative stress index (OSI) levels were calculated [[27], [28], [29], [30]].

TAS levels were measured according to published studies whereby the conversion of blue-green colored 2,2ʹ-azino-bis (3-ethylbenzthiazoline-6-sulfonic acid; ABTS) radicals to colorless reduced ABTS indicates the antioxidant level of a sample, when measured at 600 nm. The absorbance values indicate the TAS level expressed as mmol Trolox equiv/l [28].

Colorimetric methods were used to measure TOS levels as oxidation reactions cause a detectable color change [29]. The color intensity provides information about the number of oxidant molecules in the sample. H_2_O_2_ was used for calibration and the results were expressed as μmol H_2_O_2_ equiv/l [29]. The [OSI = TOS/(TAS*10)] equation was used to calculate the OSI levels of samples [30].

### **2.5** Reverse transcription-quantitative polymerase chain reaction (RT-qPCR)

The total RNA of the tissue samples was isolated using TRIzol (Invitrogen) according to the manufacturer's instructions. RNA quality was determined by a nano spectrophotometer (VWR). cDNA was synthesized from RNA (1 μg) using the iScript cDNA Synthesis kit (Bio-Rad Laboratories). The thermocycler settings for the reaction mixture were: 20 min at 46 °C, 5 min at 25 °C, and 1 min at 95 C. SYBR Green Master mix (Bio-Rad Laboratories) was used for real-time PCR amplification, and the CFX connected to the instrument (Bio-Rad) was used to detect the fluorescence signal. Each cDNA sample was analyzed in triplicates for qRT-PCR. Specific primers for HIF1α, TNF-α, and IL-6 primers were designed and GAPDH was used for normalization as the housekeeping gene. For relative and comparative quantification of gene expression, the 2^−ΔΔCt^ method was used.

### **2.6** Statistical analysis

Statistical analyses were performed with SPSS-22.00 software. One-way ANOVA followed by Duncan and LSD tests were performed to determine the statistical significance between the groups. A *p* value of *p* < 0.05 was accepted as statistically significant.

## 3 Results

### **3.1** Hematoxylin and eosin staining

**Fig. 2:**
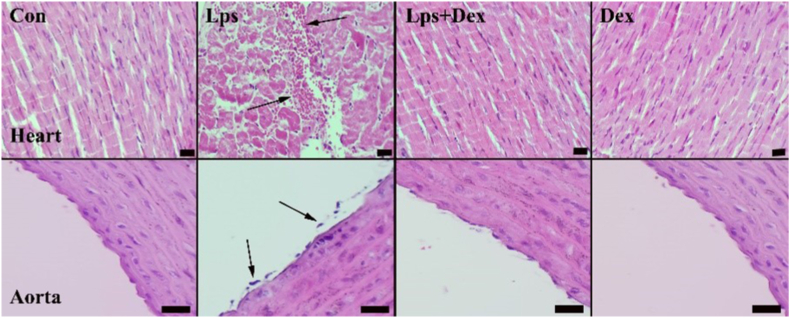
**Hematoxylin and eosin staining of rat heart and aorta: normal architectures were seen in myocardial and aortic tissues of the heart and aorta.** Myocardial hemorrhages in heart and endothelial cells depletions in aorta were seen (arrows) in the Lps group. Dex treatment recovered these pathological findings. Con = Control. Lps = Lipopolysaccharide. Dex = Dexpanthenol. Scale bars = 2 μm.

In the heart and aorta, normal tissue histology was observed in the Con and Dex groups; smooth endothelial layers were regularly observed in the aorta for both groups. The Lps group displayed myocardial cell degeneration, severe hyperemia, edema, and slight to moderate hemorrhages in heart tissue. Furthermore, endothelial cell depletion and tissue injury were observed in Lps-treated aortic samples. Dex treatment recovered the damage caused by Lps in in heart and aorta samples (Fig. 2).

### **3.2** Immunohistochemical results

**Fig. 3:**
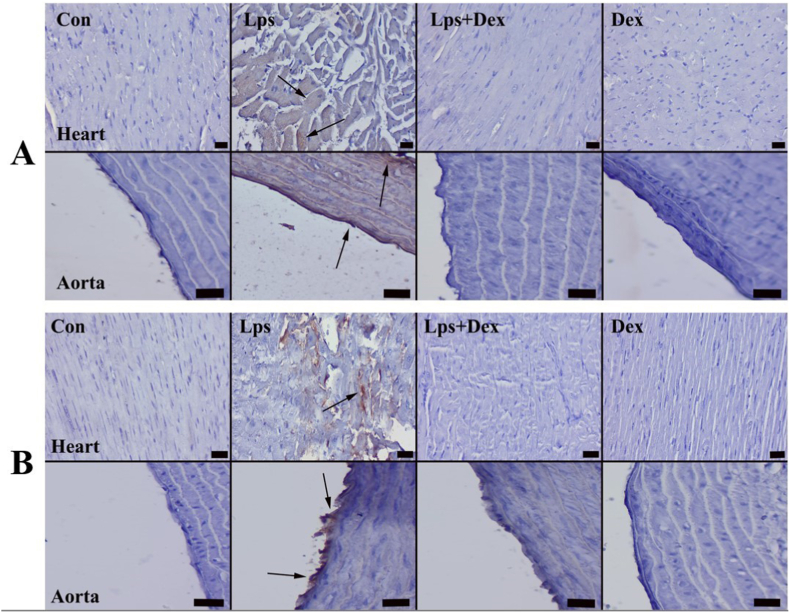
**Cas-3 and VEGF staining of rat heart and aorta.** (A) Cas-3 and (B) VEGF immunohistochemistry findings between the treatment groups. Cas-3 and VEGF expression was absent in Con and Dex groups, whereas increased expression was seen in myocardial and endothelial cells (arrows) in the Lps group, but decreased in Lps + Dex group. Con = Control. Lps = Lipopolysaccharide. Dex = Dexpanthenol. Streptavidin biotin peroxidase method, scale bars = 50 μm.

**Fig. 4:**
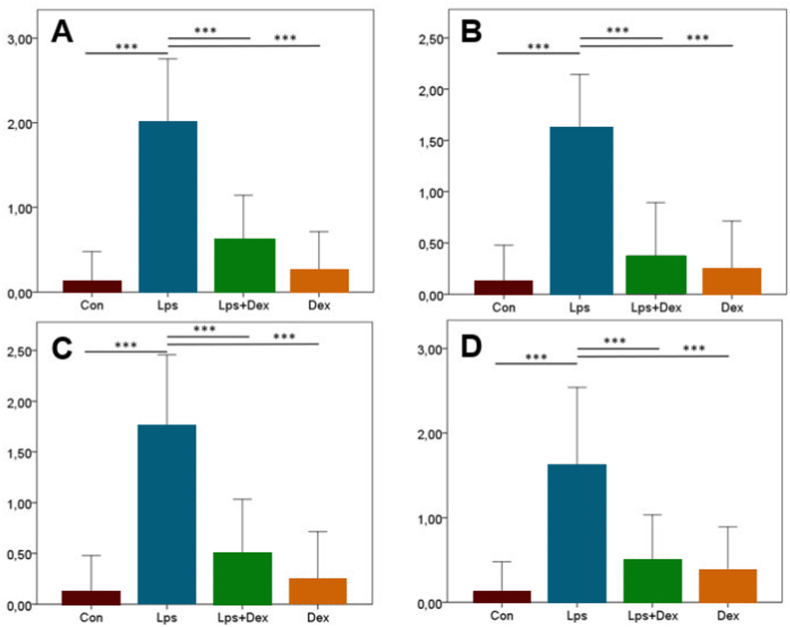
**Scoring of immunohistochemical data.** (A) Cas-3 and (B) VEGF staining scores of the heart. (C) Cas-3 and (D) VEGF staining scores of the aorta. The differences between the mean scores was represented by standard deviation (SD). P < 0.001 was considered statistically significant.

Immunohistochemical staining revealed increased expression of Cas-3 and VEGF in the Lps group, whereas Dex treatment decreased the expression of these markers. Both markers were mostly expressed in myocardial cells of the heart and endothelial cells of the aorta (Fig. 3A and B). Statistical evaluation of staining results findings is shown in Fig. 4.

Cas-3 and VEGF expression levels in the heart and aortic tissue were higher in the Lps group than in the Con group (*p* < 0.001; Fig. 4A–D). The expression levels of both markers were decreased in the Lps + Dex group compared with the Lps group (*p* < 0.001; Fig. 4A–D).

### **3.3** Biochemical results

**Fig. 5:**
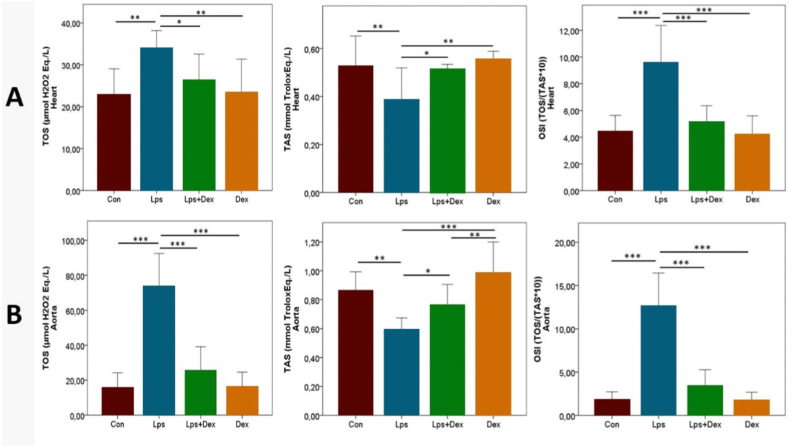
**TOS, TAS, and OSI levels.** (A) TOS, TAS, and OSI levels in heart. (B) TOS, TAS, and OSI levels in aortic tissue. Values were presented as means ± SD. TAS = Total antioxidant status. TOS = Total oxidant status. OSI=Oxidative stress index. Con = Control. Lps = Lipopolysaccharide. Dex = Dexpanthenol. * = p < 0.05. ** = p < 0.01. *** = p < 0.001.

Oxidative stress, developed with LPS application, was increased in the model group, as shown by significantly increased TOS and OSI levels in both heart and aortic tissues in the Lps group compared with the Con group (*p* < 0.01 and *p* < 0.001 in heart tissue, and *p* < 0.001 and *p* < 0.001 in aortic tissue, respectively). In the Lps + Dex group, TOS and OSI levels were significantly decreased compared with the Lps group and at near baseline levels (*p* < 0.05 and *p* < 0.001 in heart tissue, and *p* < 0.001 and *p* < 0.001 in aortic tissue, respectively; Fig. 5A–B).

The lowest TAS levels were observed in the Lps group and were significantly decreased compared with the Con group (*p* < 0.01 for heart and aortic tissue). A significant increase was found in the Lps + Dex group compared with the Lps group (*p* < 0.05 for heart and aortic tissue; Fig. 5A–B).

**Fig. 6:**
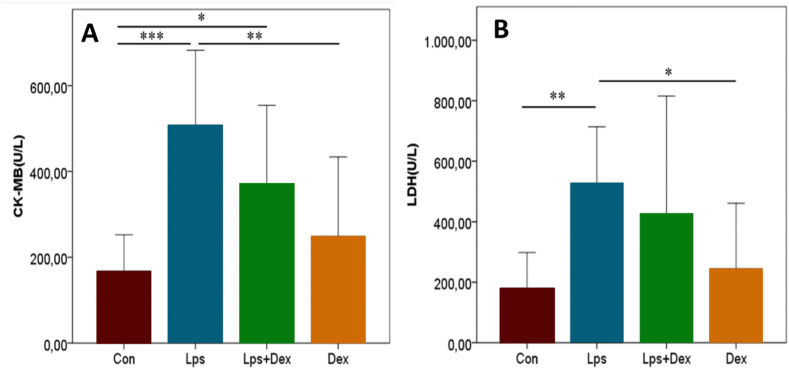
**CK-MB and LDH levels in blood.** (A) CK-MB levels. (B) LDH levels. Values were presented as means ± SD. Con = Control. Lps = Lipopolysaccharide. Dex = Dexpanthenol. CK-MB=Creatine kinase. LDH=Lactate dehydrogenase. * = p < 0.05. ** = p < 0.01. *** = p < 0.001.

Furthermore, higher levels of CK-MB and LDH levels were detected in the Lps group compared with the Con group (*p* < 0.001 and *p* < 0.01, respectively; Fig. 6A–B). Although CK-MB and LDH levels were lower in the Lps + Dex group compared with the Lps group, this was not statistically significant, yet it was double the Con levels (Fig. 6A–B).

### **3.4** RT-qPCR

**Fig. 7:**
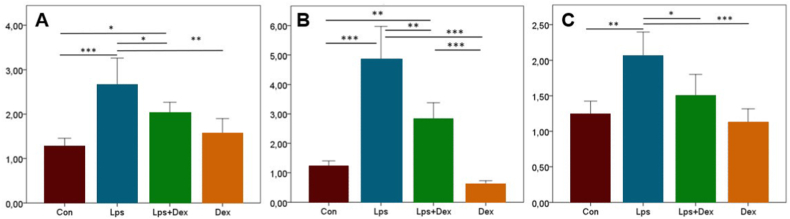
***RT-qPCR for HIF1α, IL-6, and TNF-α.****(A) HIF1α, (B) IL-6, and (C) TNF-α mRNA expression levels in rat heart tissue. Con = Control*. *Lps = Lipopolysaccharide*. *Dex = Dexpanthenol*. *HIF1α = Hypoxia-inducible factor 1 α*. *IL-6 = Interleukin-6*. TNF-α = Tumor necrosis factor-α. * = p < 0.05. ** = p < 0.01. *** = p < 0.001. Values were presented as means ± SD.

The relative mRNA expression of HIF1α was significantly higher in the Lps group compared with the Con group, and reduced with the addition of Dex group, but remained double the level of the Con group (*p* < 0.001, *p* < 0.05, respectively; Fig. 7A).

The mRNA expression of IL-6 was significantly higher in the Lps group compared with the Con group, while expression was considerably reduced in the Lps + Dex group in comparison to the Lps group (*p* < 0.001, *p* < 0.01, respectively; Fig. 7B).

Following the same pattern, a higher TNF-α mRNA expression was found in the Lps group compared with the Con group, which was subsequently reduced with the addition of Dex when compared with the Lps group (*p* < 0.001, *p* < 0.05, respectively; Fig. 7C).

## 4 Discussion

This study has shown that Dex, which is reported to have anti-inflammatory and antioxidative properties, can have protective effects against myocardial and epithelial injury triggered by Lps. Furthermore, our data suggests that the protective effect of Dex might be mediated by the IL-6/HIF1α/VEGF pathway, which has not yet been revealed in similar studies of the protective properties of Dex.

Our histopathological analyses showed damage caused by inflammation manifested as hyperaemic areas, edema, and hemorrhages in the heart and endothelial tissue of the Lps-administered rats, which is consistent with previous studies and support our Lps-induced model of inflammation [[31], [32]]. We observed clear histological changes and damage induced by Lps and a visual reduction of damage when the rats were given Dex. VEGF staining was detected in areas of inflammation that we corroborated with increased IL-6 and TNF-α mRNA expression. The addition of Dex countered Lps-induced inflammatory changes that we observed, as the Lps-mediated increased mRNA expression llevels of TNF-α and IL-6 were reduced (but not to baseline levels), as suggested in previous studies [33].

The acute phase reactants IL-6 and TNF-α can activate various intracellular pathways and cellular damage mechanisms. These mechanisms that trigger each other can cause the damage to progress rapidly and progressively. Inflammation can induce oxidative stress and vice versa. Here, oxidative stress was observed by increased TOS and OSI values in the Lps group, but was restored by Dex treatment. Similarly, the antioxidative effects of Dex were demonstrated by decreased TOS and OSI levels in an isoproterenol-induced cardiac injury model [34]. Our findings highlight Dex's ability to combat increased oxidative stress due to Lps.

In contrast to the significant decrease in TOS values, Dex-induced TAS elevation in the current study was not statistically significant. Lps showed a significant increase of TOS and OSI levels, which were reduced to near baseline levels with the addition of Dex. Similarly, where Lps reduced TAS, Dex could restore expression to near Con levels. Considering the results obtained for the expression of oxidative stress markers, the antioxidant activity of Dex can be interpreted to have direct and indirect effects. Dex might have directly increased TAS levels or promoted the release of an antioxidative product by reducing oxidative stress secondary to inflammation. The lack of a sufficient increase in TAS values in the Dex group compared with the Con group might be because the drug reduces oxidative stress secondary to its anti-inflammatory properties. In addition, the non-significant increase of TAS levels compared with Con, could be due to a lower drug dose or shorter treatment period than what is required to synthesize the antioxidative enzymes. Dose and duration studies should be conducted to explain the reasons why Dex does not cause a significant increase in TAS value.

In the case of impaired perfusion, oxidative stress and inflammation could drive some adaptive cellular changes. Some intracellular pathways are activated to supply the cells responsible for the tissue repair to the damaged area. The IL-6/HIF1α/VEGF pathway is activated in the presence of inflammation. The increase in HIF1α/VEGF gene expressions, largely secondary to the incremental change in IL-6 level found in our findings, supports this theory [[35], [36]]. Activation of this pathway, which is responsible for the increase in vascularity caused by inflammation in tissues, is considered normal up to a certain threshold. Here we observed that Dex decreased HIF1α/VEGF expressions as part of an anti-inflammatory process, by decreasing IL-6 and TNF-α levels.

Perfusion impairment in the distal tissues due to damage to vascular structures might lead to apoptosis. Immunohistochemically, we observed that Lps increased Cas-3 staining and that Dex treatment suppressed apoptosis, secondary to its antioxidative and anti-inflammatory effects. The increased expression of HIF1α under hypoxic conditions was similar to Cas-3 levels. In another study, Dex was shown to reduce Cas-3 expression levels in a carbon tetrachloride-induced myocardial toxicity model [37]. Our findings show the anti-apoptotic effect of Dex on heart tissue.

Although not specific to the heart, increases in CK-MB levels are considered an indicator of acute cardiac injury [38]. Similarly, for cardiac cell damage increased LDH levels are an expected result [39]. CK-MB values begin to rise 4–9 h after cardiac injury, while the increase in Lps occurs after 12–24 h following myocardial pathologies [[40], [41]]. However, although few in number, there are publications showing insignificant increases in LDH levels starting 4–6 h after Lps application [[42], [43]]. Our findings contribute to the debate on whether there is a significant increase in LDH levels in the first 6 h after Lps injection by showing significant elevations in the Lps-treated group. Of note, Dex alone showed increased CK-MB and LDH compared with Con, although not significantly, but when Dex was given to Lps-treated mice there was a reduction in CK-MB and LDH levels. This non-significant decrease in CK-MB and LDH levels by Dex requires further studies with longer treatment durations to better understand the effects that Dex has on normal tissue and how Lps-induced inflammation changes the Dex response. This has important implications for Dex treatment and when and under which conditions Dex would be most effective.

To summarize, we have shown that Lps given to rats causes tissue damage by means of inflammation that subsequently causes oxidative stress and apoptosis. In contrast, Dex decreased tissue damage by anti-inflammatory and antioxidant mechanisms. Our data highlights that Dex affects IL-6/HIF1α/VEGF signalling, but the exact mechanism for tissue protection remains unclear given the numerous factors that could also be affected, such as cytokines and other overlapping signalling pathways. HIF-1α expression might be declined due to decreased inflammation and oxidative stress. As TAS levels were not significantly increased by Dex, it is more possible that the anti-inflammatory effect of Dex is more prominent than the antioxidant effect. To clearly state what is the cause and result, related pathways and cytokines should be fully evaluated.

Our study has some limitations. First, we could not perform some analyses such as measurement of blood pressure and electrocardiography or echocardiography before and after drug administrations to monitor the cardiac damage clinically. Demonstrating the cardiac functions in relation to Lps and Dex injections would have allowed us to monitor the real-time effects on the heart and recovery of the rats. Second, we measured LDH levels to show cardiac damage. LDH is present in all the tissues involved in glycolysis and is mediated by five different isoenzymes. To state more definitively that LDH is specific to cardiac damage, specific isoenzyme levels (LDH-1 and LDH-2) should be evaluated [41]. Third, oxidative changes in Lps-treated tissues and the ameliorative effects of Dex were shown by non-conventional methods. Given better resources, we would have investigated oxidative changes more thoroughly; for example, protein damage, DNA damage, enzymatic antioxidants, glutathione, and so on. Although these methods are valid and used in many studies, it would be better to verify the oxidative stress by conventional methods like Western blot or immunofluorescence. If heart cell lines were available to us, we would screen the IL-6/HIF1α/VEGF and related pathways using siRNA to elucidate the direct effectors of the changes that we observed. Lastly, our study had an acute design that evaluated the short-term effects of Dex, but not the chronic effects. As sepsis has acute and chronic phases, further studies are needed to establish the chronic effects of the treatment agents.

## 5 Conclusion

Our study has shown that Dex has a protective effect on Lps-induced cardiac and endothelial damage. Dex decreased inflammation, reduced oxidative stress, and apoptosis in rat heart tissue. Significant decreases in HIF1α, IL-6, TNFα, and VEGF levels were responsible for the protective mechanism of Dex. Understanding this mechanism will be helpful for use Dex in other indications. Indeed, there is a need for further investigations to understand the mechanism of action of Dex with other signalling pathways and cellular responses related to cardiac and endothelial damage caused by inflammation. As this is an experimental model, it is not possible to show the exact effects of Dex in humans; however, inferences can be made from our findings, which can be significant for future human studies. Given the easy accessibility and relatively cheap nature in addition to its anti-inflammatory and antioxidant effects, Dex can be added as an adjuvant to the existing treatment in patients diagnosed with sepsis.

## Data availability statement

Data associated with the study has not been deposited into a publicly available repository and data will be made available on request.

## Human and animal rights

The authors declare that the work described has been carried out in accordance with the Declaration of Helsinki of the World Medical Association revised in 2013 for experiments involving humans as well as in accordance with the EU Directive 2010/63/EU for animal experiments.

## Informed consent and patient details

Not applicable.

## Funding

This work has been supported by the Scientific Research Project Unit of Suleyman Demirel University to supply materials and acquire, analyze, and interpret data [Project code TSG-2020-8134]. Süleyman Demirel University, Isparta, Turkey.

## CRediT authorship contribution statement

**Mustafa Soner Ozcan:** Writing - review & editing, Writing - original draft, Methodology, Investigation, Formal analysis, Data curation, Conceptualization. **Mehtap Savran:** Writing - original draft, Resources, Methodology, Investigation, Data curation, Conceptualization. **Duygu Kumbul Doguc:** Writing - review & editing, Visualization, Formal analysis, Data curation. **Hatice Kubra Dogan:** Writing - review & editing, Visualization, Resources, Investigation, Formal analysis, Data curation. **Melike Altintas:** Writing - review & editing, Visualization, Resources, Investigation, Data curation. **Samet Cosan:** Writing - review & editing, Resources, Investigation, Data curation.

## Declaration of competing interest

The authors declare the following financial interests/personal relationships which may be considered as potential competing interests:Mustafa Soner OZCAN reports financial support was provided by The Scientific Research Projects Coordination Unit of Suleyman Demirel University (TSG-2020-8134).
